# Interferon-γ promotes gastric lymphoid follicle formation but not gastritis in *Helicobacter*-infected BALB/c mice

**DOI:** 10.1186/s13099-016-0142-0

**Published:** 2016-11-21

**Authors:** Michelle Chonwerawong, Patrick Avé, Michel Huerre, Richard L. Ferrero

**Affiliations:** 1 Centre for Innate Immunity and Infectious Diseases, Hudson Institute of Medical Research, Monash University, 27-31 Wright Street, Clayton, VIC 3123 Australia; 2 Unité de Recherche et d’Expertise Histotechnologie et Pathologie, Institut Pasteur, 25-28 Rue du Dr Roux, 75724 Paris, France; 3 Department of Microbiology, Monash University, Clayton, VIC Australia; 4 Unité de Histopathologie Humaine et Modèles Animaux, Institut Pasteur, 25-28 Rue du Dr Roux, 75724 Paris, France; 5 Département de Pathologie, Institut Curie, 26 Rue d’Ulm, 75248 Paris, France

**Keywords:** *Helicobacter*, Interferon-gamma, Lymphoid follicle, Mucosa-associated lymphoid tissue, MALT lymphoma, BALB/c, T helper response, Gastric inflammation

## Abstract

**Background:**

Mouse infection studies have shown that interferon-γ (IFN-γ), a T helper 1 (Th1) cytokine, is required for the development of severe pathology induced by chronic *Helicobacter* infection. This finding is largely based on studies performed using mice that have polarised Th1 responses i.e. C57BL/6 animals. The current work aims to investigate the role of IFN-γ in *Helicobacter*-induced inflammation in BALB/c mice which have Th2-polarised immune responses.

**Results:**

At 7 months post-infection with *Helicobacter felis,* IFN-γ deficiency in BALB/c mice had no significant effect on *H. felis* colonisation levels in the gastric mucosa, nor on humoral responses, or gastritis severity. *Ifng* ^−/−^ animals with chronic *H. felis* infection did, however, develop significantly fewer lymphoid follicle lesions, as well as increased IL-4 splenocyte responses, when compared with infected *Ifng* ^+/+^ mice (P = 0.015 and P = 0.0004, respectively).

**Conclusions:**

The work shows that in mice on a BALB/c background, IFN-γ is not required for bacterial clearance, antibody responses, nor gastric inflammation. Conversely, IFN-γ appears to play a role in the development of gastric lymphoid follicles, which are precursor lesions to mucosa-associated lymphoid tissue (MALT) lymphoma. This study highlights the importance of mouse host background on the susceptibility to *Helicobacter*-induced pathologies.

## Background

The prolonged immune responses generated during *Helicobacter pylori* infection in humans drive the development of diseases that vary in severity, ranging from peptic ulcers to gastric adenocarcinomas and lymphomas [1]. One of the key soluble mediators induced during *Helicobacter* infection is interferon-γ (IFN-γ), a pro-inflammatory cytokine that contributes to gastric inflammation and is a hallmark of T helper (Th) type 1 responses [2–4].

*Helicobacter* infection has been reported to induce both Th1 and Th2 responses [5–7]. Th1 responses are characterised by immune cell production of IFN-γ and interleukin-12 (IL-12) [8, 9], whereas Th2 responses are associated with strong humoral responses and the production of cytokines, such as IL-4 and IL-13 [10–12]. Intraepithelial lymphocytes have been shown to be a major source of IFN-γ and IL-4 production within the gastric mucosa of *Helicobacter*-infected murine and human hosts [2, 3, 5, 13]. High levels of IFN-γ production have been observed in both gastric CD4^+^ T cells and splenocyte cultures from infected hosts, thereby implicating severe inflammation with skewed Th1 responses [5, 9]. IFN-γ was also reported to play a role in bacterial clearance [4, 13].

Mice that have been experimentally infected with the feline/canine *Helicobacter* sp., *Helicobacter felis*, develop many aspects of the pathological changes observed in *H. pylori*-infected humans, including the progression from chronic gastritis to cancer, commonly known as the “Correa model” [1, 14–18]. Although the type(s) and severity of pathology observed in *Helicobacter* infection models are known to be influenced by the genetic background of the mice [3, 19, 20], little is still known regarding the role of host factors in *Helicobacter*-induced immunopathology. Previously, we proposed that the default Th phenotypes of mice may be one factor contributing to the different types of pathology seen in animals [21].

Wild-type C57BL/6 mice are relatively susceptible to *Helicobacter* infection and develop severe atrophic gastritis and pre-neoplastic lesions [1, 19, 22]. In contrast, mice on a BALB/c background appear to be more resistant to *Helicobacter* infection [20, 22, 23] and usually develop little to no gastritis in the first few months post-infection [16, 22, 24]. However, after long-term infection (i.e. 18–24 months) with either *H. pylori* or *H. felis*, BALB/c mice develop structured gastric lymphoid follicles resembling mucosa-associated lymphoid tissue (MALT) lymphoma in humans [16, 20, 22, 25].

Many of the *Helicobacter* infection studies in the literature have used mice on a C57BL/6 background. These animals have Th1-polarised immune responses [7, 9, 26]. Given that IFN-γ is a key mediator of these responses [7, 9, 26], we sought to determine whether IFN-γ is required for *Helicobacter*-induced inflammation in mice with Th2-biased responses. To address this question, we used *H. felis* to experimentally infect *Ifng* ^+/+^ and *Ifng* ^−/−^ mice on a BALB/c background which have Th2-polarised immune responses. From these experiments, we were able to show that IFN-γ plays no role in cellular or humoral immune responses to chronic *H. felis* infection in BALB/c mice. Importantly, however, the absence of IFN-γ in BALB/c animals resulted in a significant reduction in lymphoid aggregate formation, when compared with infected *Ifng* ^+/+^ mice. These results demonstrate the influence of the mouse genetic background on the pathology observed in *Helicobacter* infection models.

## Methods

### Animals

*Ifng* ^+/+^ and *Ifng* ^−/−^ BALB/c mice (The Jackson Laboratory, Bar Harbor, ME, USA) were housed in polycarbonate cages in isolators and fed a commercial pellet diet with water. Groups of mice (*n* = 8–10) were inoculated with >10^6^ *H. felis* CS1 bacteria or given broth alone [21]. At 7 months post-infection, mice were sacrificed. All animal experimentation was performed in accordance with institutional guidelines, prescribed by the committee of Hygiène Sécurité et Protection de L’Environnement (Institut Pasteur), according to French Law 87-848.

### Samples

Gastric tissues from mice were divided into two sections, each containing the antrum and body mucosa and used to determine *H. felis* colonisation, gastric antibody levels and histology studies [21]. Sera were collected in Sarstedt microtubes (Sarstedt) and stored at −20 °C for further testing. Gastric secretions were collected as described previously [27]. Briefly, mouse stomachs were opened along the greater curvature and the contents released into phosphate-buffered saline (pH 7.4) in six-well tissue culture plates (Falcon, Becton–Dickinson Labware). Protease inhibitors were added to samples and stored at −20 °C. Spleens were collected in RPMI 1640 medium (Gibco) containing 5% (vol/vol) foetal calf serum, 200 mM l-glutamine, 10,000 IU/mL penicillin and 10,000 mg/mL streptomycin (RPMI complete medium; all solutions from Life Technologies™) [27].

### Histology

Formalin-fixed gastric tissue segments were stained with Harris’ Hematoxylin and Eosin or Giemsa and then assessed blind for histopathological changes [28] and *Helicobacter felis* colonisation levels [29], respectively. Briefly, inflammatory scores for polymorphoneutrophils (PMNs) and lymphocytes were graded according to a previously described six-point scheme ([21, 30]; Table 1). Lymphoid aggregates were graded according to the actual numbers of glands affected. Since *H. felis* does not normally form isolated colonies on culture plates, we assessed the proportion of Giemsa-stained fields according to the following scale: 0, none; 1, 1–10; 2, 10–100; and 3, >100 bacteria [29]. A total of 34–82 fields were graded accordingly per mouse. Cumulative scores for each mouse were calculated according to the formula: cumulative score = n_0_/t + n_1_/t + n_2_/t + n_3_/t, where n_0_, n_1_, n_2_ and n_3_ = the numbers of fields with respective scores of 0, 1, 2 or 3. t = total numbers of fields assessed.

**Table 1:** Histological scoring scheme to grade PMN and lymphocyte inflammatory scores

Score	Criteria	Definition of criteria
0	No inflammation	Absence of immune cells in the mucosa
1	Mild^a^ multifocal	Scattered clumps of two or three immune cells
2	Mild widespread OR moderate^b^ multifocal	Mild widespread = widespread scattering of two or three immune cells across most of the antrum or fundus; moderate multifocal = larger clumps of immune cells seen in a few fields per region
3	Mild widespread AND moderate OR severe^c^ multifocal	As per 2. Severe multifocal = large infiltrations of immune cells across the whole width of the mucosa
4	Moderate widespread	Large clumps of immune cells seen throughout the whole width of mucosa
5	Moderate widespread and severe multifocal	As per 4 but also with areas of dense concentrations of immune cells
6	Severe widespread	Dense sheets of immune cells throughout the mucosa

^a^Mild inflammation was defined as an influx of inflammatory cells in the basal region of the mucosa

^b^Moderate inflammation describes inflammatory cells extending up to the mid-region of the mucosa

^c^Severe inflammation describes immune cell infiltration throughout the full thickness of the mucosa

### Enzyme-linked immunoassay (ELISA)

Total and *H. felis*-specific gastric (IgA, IgG) and serum (IgG1, IgG2a) antibodies were detected by ELISA [27]. Ninety-six well Nunc Maxisorp plates (Nunc) were coated with either goat anti-mouse IgA (SouthernBiotech) or anti-mouse IgG antibodies (GE Healthcare Life Sciences; 100 ng/well), or sonicated *H. felis* extracts (25 µg protein/well). Serum and gastric lavage samples were diluted 1:1000 and 1:100, respectively. Biotinylated goat anti-mouse antibodies and streptavidin-peroxidase conjugate (GE Healthcare Life Sciences) were used to detect total or antigen-specific immunoglobulins. Immune complexes were detected as described previously [21]. Total antibody levels were determined using standard curves derived from purified polyclonal IgG and monoclonal IgA mouse antibodies (Sigma). *Helicobacter felis*-specific IgG1 and IgG2c were determined from absorbance readings at 405 nm (test) and 492 nm (reference) wavelengths.

### Splenocyte responses

To isolate single cell suspensions, tissues were homogenised using Teflon grinders and recovered by centrifugation in Ficoll-Paque solution (GE Healthcare Life Sciences), as described previously [27]. Splenocytes were recovered in RPMI complete medium and seeded at 2 × 10^6^ cells/mL in 24-well tissue cultures. Cells were left untreated or stimulated with an anti-CD3 antibody (5 µg/mL; BD Pharmingen), then incubated for 3 days at 37 °C in 5% CO_2_. IL-4 and IFN-γ levels in culture supernatants were tested by sandwich ELISA (BD Pharmingen™).

### Statistical analyses

All analyses were performed using Graphpad Prism version 6.0c. Data were analysed by either the Mann–Whitney test, one- or two-way analysis of variance (ANOVA), as appropriate. Presented are the median values or means and standard error of the mean (SEM). Differences were considered significant when P < 0.05.

## Results

### IFN-γ is not required to control *H. felis* colonisation in BALB/c mice

**Fig. 1:**
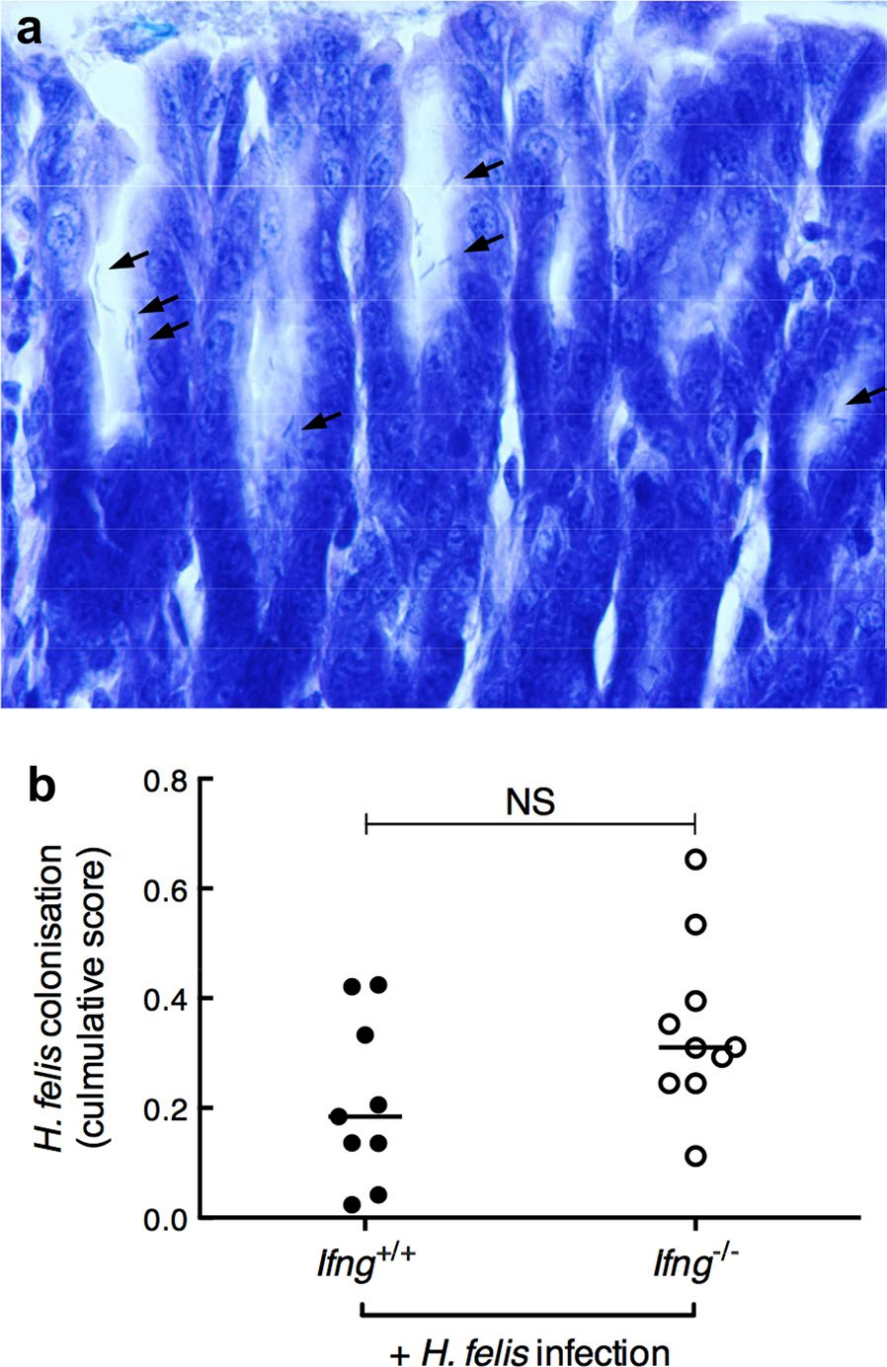
*H. felis* colonisation levels were similar in *Ifng* ^+/+^ and *Ifng* ^−/−^ BALB/c mice. **a** Giemsa-stained gastric section showing glands lined with *H. felis* bacteria, indicated by *arrows* (magnification ×40). **b** *H. felis* colonisation levels, as determined from the assessment of Giemsa-stained sections, are presented as cumulative scores (see “Methods” section). Values for individual mice are presented. *Horizontal bars* correspond to the median value. Mann–Whitney test: *NS* not significant

To determine whether the absence of IFN-γ in BALB/c mice may have an effect on *Helicobacter* bacterial burden, we infected *Ifng* ^+/+^ and *Ifng* ^−/−^ mice on a BALB/c background with *H. felis*. Control *Ifng* ^+/+^ mice received broth medium alone. As *H. felis* does not normally form isolated colonies on culture plates, colonisation levels were assessed by examination of Giemsa-stained fields [29] (Fig. 1a). All *H. felis*-challenged mice (n = 19) became infected (data not shown). However, no significant differences in *H. felis* colonisation levels were observed between *Ifng* ^+/+^ and *Ifng* ^−/−^ mice (Fig. 1b), thus showing that IFN-γ is not required to control *H. felis* colonisation in the gastric mucosa of BALB/c animals.

### *Ifng*^−/−^ mice display normal gastric and serum antibody responses to *H. felis* infection

**Fig. 2:**
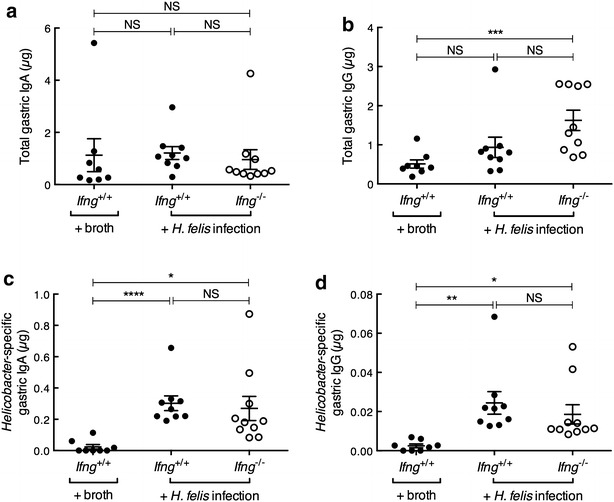
Gastric antibody levels in *Ifng* ^+/+^ and *Ifng* ^−/−^ BALB/c mice. Total (**a**) IgA and (**b**) IgG and *Helicobacter*-specific (**c**) IgA and (**d**) IgG antibodies. Values for individual mice are presented and were determined from triplicate measurements. *Horizontal bars* correspond to the mean ± SEM. One-way ANOVA: *NS* not significant; *P = 0.05. **P = 0.01; ***P = 0.008; ****P = 0.005

**Fig. 3:**
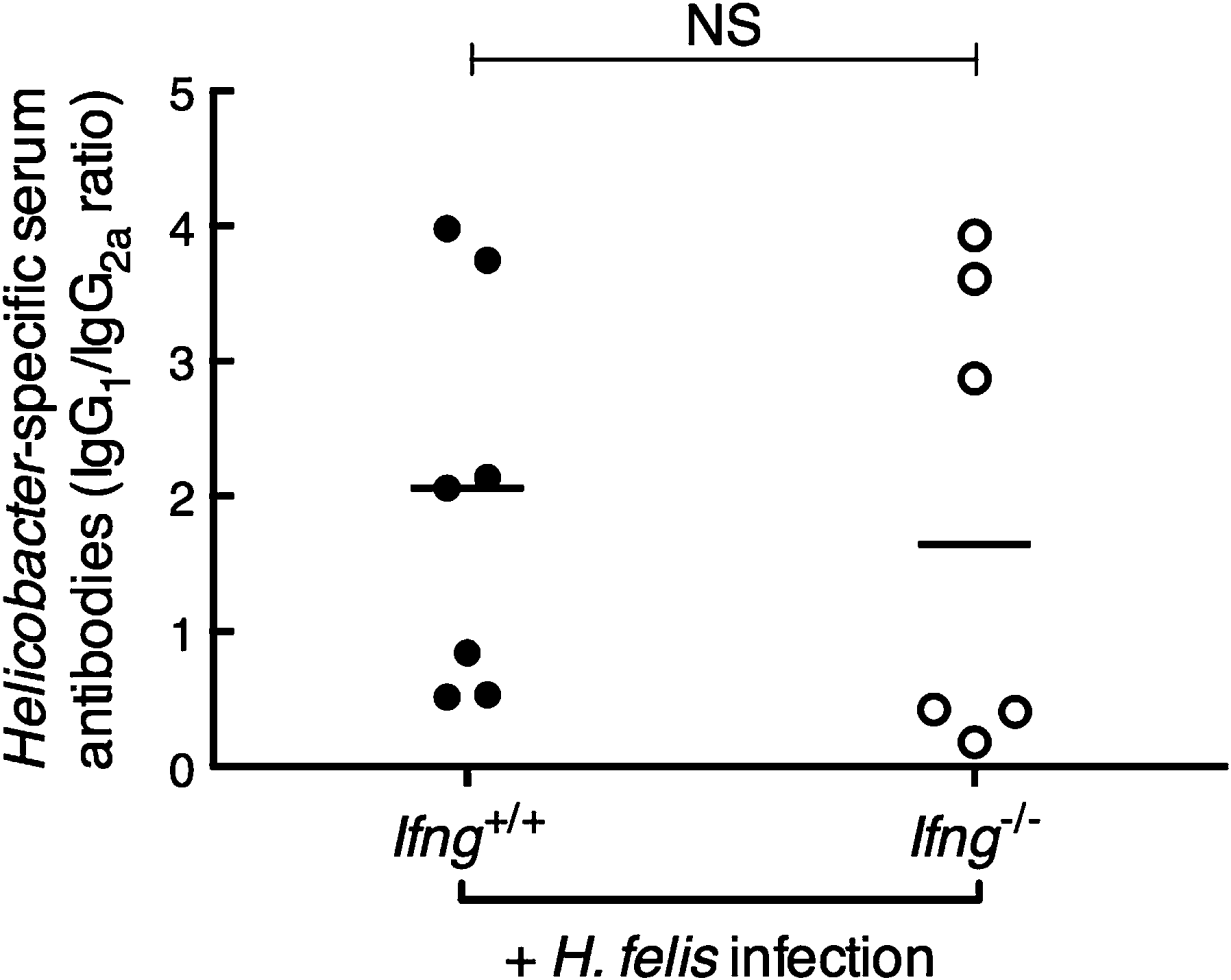
*Helicobacter*-specific serum IgG1 and IgG2a responses were similar in *Ifng* ^+/+^ and *Ifng* ^−/−^ BALB/c mice. Values for individual mice are presented as ratios calculated from paired serum samples (n = 6–7/group). *Horizontal bars* correspond to the median value. Mann–Whitney test: *NS* not significant

IFN-γ is known to play a role in mucosal antibody responses and to promote B cell isotype switching [31–34]. IFN-γ deficiency in BALB/c mice, however, had no effect on total IgA and IgG levels in gastric juices (Fig. 2). Furthermore, no differences were observed in either *Helicobacter*-specific IgA and IgG levels in the gastric compartment, nor in serum IgG1 and IgG2a levels, which are indicative of Th2- and Th1-responses, respectively (Fig. 3). These results indicate that IFN-γ does not regulate mucosal antibody responses in BALB/c animals during chronic *H. felis* infection.

### *Ifng*^−/−^ BALB/c mice exhibit normal levels of gastritis but reduced lymphoid follicle formation in response to chronic *H. felis* infection

**Fig. 4:**
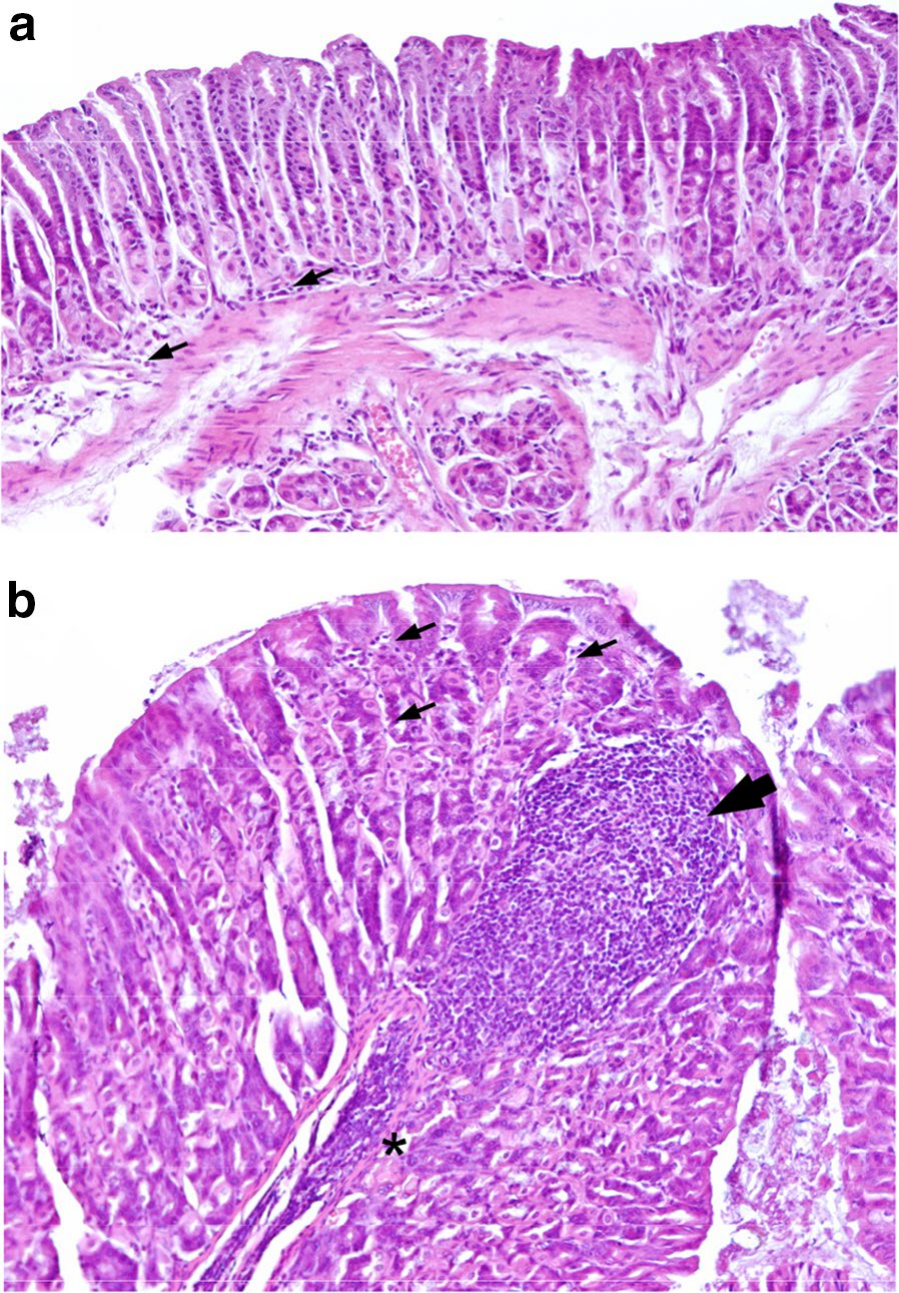
Chronic *H. felis* infection promoted mucosal inflammation and lymphoid cell formation in *Ifng* ^+/+^ BALB/c mice when compared with uninfected animals. Gastric tissue sections from (**a**) uninfected and (**b**) *H. felis*-infected *Ifng* ^+/+^ BALB/c mice.* Small arrows* show immune cells (mainly lymphocytes) present either in the (**a**) submucosa or (**b**) between the glands in the mucosal region. Massive immune cell infiltration of the submucosa (*asterisk*) and mucosal lymphoid cell formation (*arrowhead*) in an *H. felis*-infected mouse. Haematoxylin and Eosin stain; magnification ×10

**Fig. 5:**
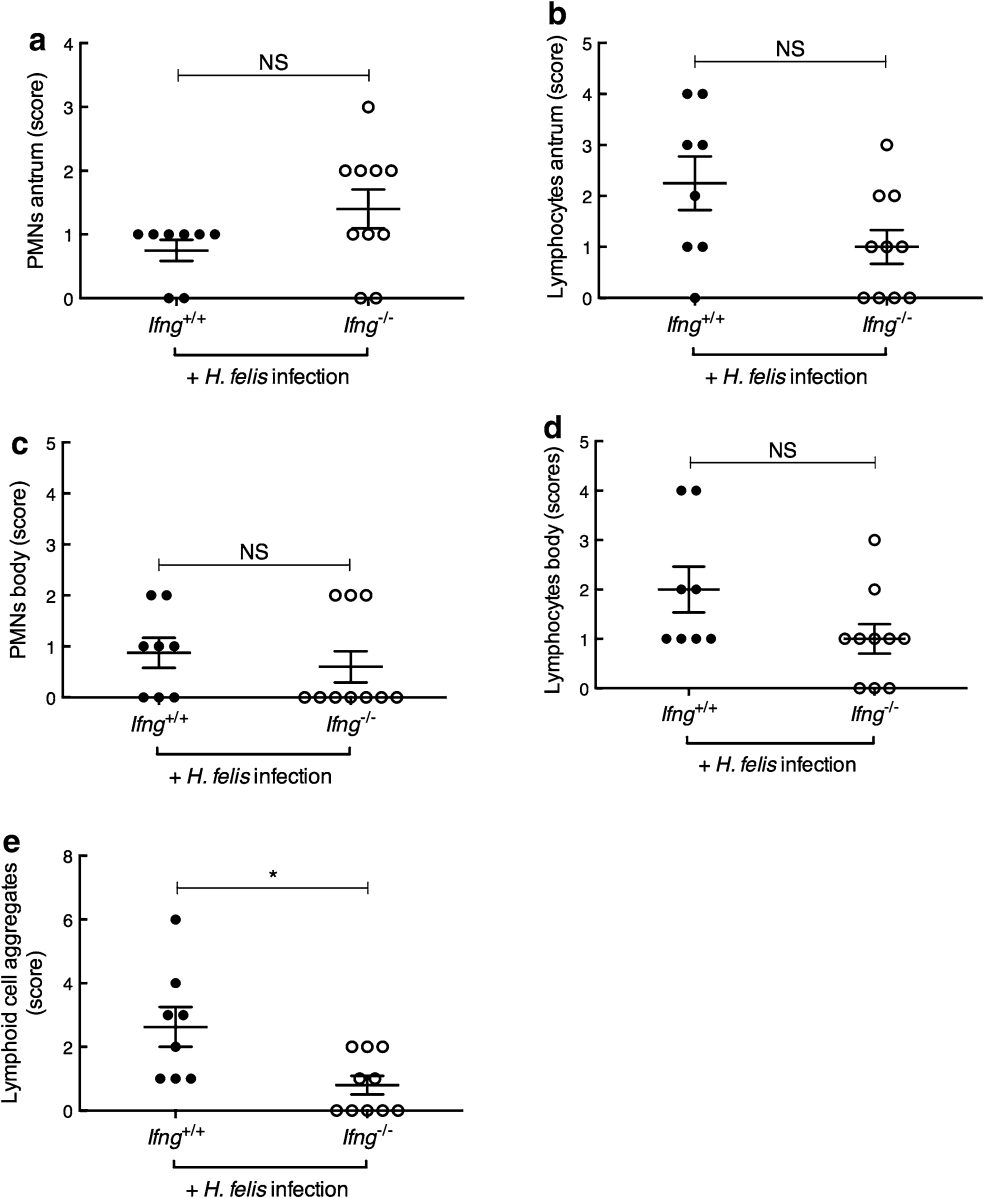
Chronic *H. felis* infection induced similar levels of gastritis but less lymphoid aggregate formation in *Ifng* ^−/−^ BALB/c mice than in infected *Ifng* ^+/+^ animals. The levels of PMN and lymphocyte infiltration in the **a**, **b** antrum and **c**, **d** body of the gastric mucosa were scored blind according to the grading scheme in the “Methods” section. **e** Lymphoid aggregate numbers were assessed over the entirety of gastric tissue sections. Histopathological scores for individual mice are presented. One-way ANOVA: *NS* not significant; *P = 0.015

We next sought to investigate whether IFN-γ may be important for histopathological changes in the gastric tissues of *H. felis*-infected mice on a Th2-biased BALB/c background. As controls, uninfected *Ifng* ^−/−^ BALB/c mice were also assessed histologically but showed a normal gland architecture (data not shown), with only some immune cells occasionally present in the submucosal region of the stomach (Fig. 4). In contrast, infected *Ifng* ^+/+^ BALB/c mice displayed general thickening of the glandular tissues, immune cell infiltration of the mucosa and invasion of mucosal areas by large lymphoid cell aggregates (Fig. 4). The levels of PMN and mononuclear cell infiltration did not significantly differ between *Ifng* ^+/+^ and *Ifng* ^−/−^ mice (Fig. 5). Gland atrophy was not detected in tissues of infected or uninfected mice (data not shown). Interestingly, *Ifng* ^−/−^ mice developed significantly fewer lymphoid aggregates within the gastric mucosa when compared with infected *Ifng* ^+/+^ animals (Fig. 5; P = 0.015). These findings indicate that IFN-γ does not play a major role in the gastritis induced by chronic *H. felis* infection in BALB/c mice. In contrast, this cytokine is likely to be important for gastric lymphoid tissue formation, a precursor to MALT lymphoma.

### IFN-γ deficiency in BALB/c mice is associated with increased IL-4 splenocyte responses following chronic *H. felis* infection

**Fig. 6:**
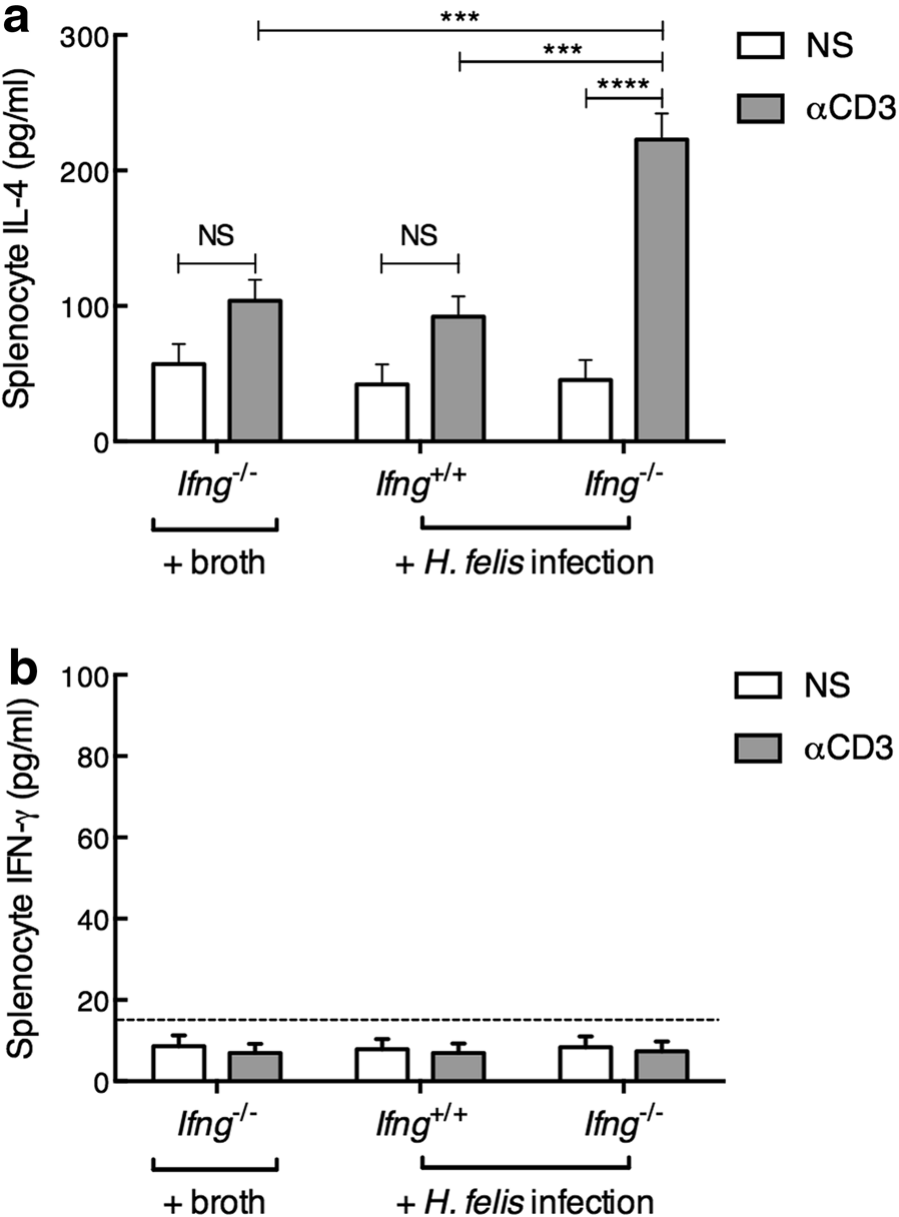
Increased IL-4 production in response to stimulation by splenocytes from *H. felis*-infected *Ifng* ^−/−^ mice when compared with infected *Ifng* ^+/+^ BALB/c animals. The levels of **a** IL-4 and **b** IFN-γ production were determined in splenocyte cultures that were either not stimulated (NS) or stimulated with anti-CD3 antibody (αCD3, 5 µg/mL). Data are presented as the mean value (±SEM) determined from triplicate measurements for 8–10 mice/group. Two-way ANOVA: *NS* not significant; ***P = 0.004; ****P < 0.0001

As BALB/c mice have a polarised Th2 profile [21, 35], we speculated that the absence of *Ifng* ^−/−^ would result in enhanced Th2-type cytokine responses to chronic *H. felis* infection in these mice. Indeed, anti-CD3 treatment of splenocyte cultures from *H. felis*-infected *Ifng* ^−/−^ mice induced significantly increased levels of IL-4 production when compared with untreated splenocytes from these mice (Fig. 6; P < 0.0001). Most importantly, stimulated splenocytes from *H. felis*-infected *Ifng* ^−/−^ mice produced significantly more IL-4 when compared with splenocytes from either *H. felis*-infected *Ifng* ^+/+^ animals or uninfected *Ifng* ^−/−^ mice (Fig. 6; P = 0.0004). Anti-CD3-treatment did not induce detectable levels of IFN-γ production in *Ifng* ^−/−^ splenocytes (Fig. 6). Taken together, the data are consistent with the Th2-polarised immune response of BALB/c mice [21, 35] and, moreover, show that chronic *H. felis* infection promotes an enhanced production by splenic immune cells of IL-4, a Th2 cytokine. The skewed Th2 response may influence the degree or development of gastric diseases induced by chronic *H. felis* infection.

## Discussion

IFN-γ has been reported to be an important mediator of the immunopathology induced by chronic *Helicobacter* infection [4, 7, 9, 13, 36, 37] and may also be important for *Helicobacter* clearance, but the findings are not conclusive [4, 10, 13, 23]. Importantly, these observations were largely based on studies performed in C57BL/6 mice which have default Th1 type immune responses. We now show in a Th2-polarised mouse model that IFN-γ was not required for host defence against experimental *Helicobacter* infection, nor for the induction of humoral immune responses or gastritis. Interestingly, however, IFN-γ was found to be important for lymphoid aggregate formation. This work highlights the impact of the mouse genetic background on host immune responses and the accompanying immunopathology induced by chronic *Helicobacter* infection.

The influence of mouse genetic background on *Helicobacter*-induced immunopathology was first described two decades ago [3, 24], yet our understanding of the reasons for varying disease severity between mouse strains remains poor. In humans, it was reported that host factors, such as cytokine gene polymorphisms, can influence disease outcome to *H. pylori* infection [38]. Of particular relevance here, Th1/Th2 profiles in human hosts were shown to have an impact on the immunopathogenesis of *H. pylori* infection, with Th1 cytokines associated with stronger cellular responses and early stages of gastric cancer, whereas Th2 polarised responses mediated humoral immunity and advanced stages of carcinogenesis [39]. There is clear evidence that the default T helper cell responses of mouse strains contribute to inflammation severity [13, 21, 26, 36, 40, 41]. However, other immunological characteristics intrinsic to specific mouse strains, such as their major histocompatibility complex haplotype, are also likely to be important [3, 20, 21, 23, 30, 35, 42, 43].

*Helicobacter*-infected C57BL/6 mice develop more intense gastric inflammation than BALB/c animals [3, 9, 20, 22, 24], with disease severity correlating with higher levels of IFN-γ production [3, 4, 9]. Interestingly, *H. pylori* infection in *Il4* ^−/−^ mice on a C57BL/6 background resulted in more severe gastritis and higher levels of IFN-γ production by stimulated splenocytes [9]. It has been suggested that the ability of IFN-γ to promote macrophage secretion of pro-inflammatory cytokines, and/or to downregulate the production of anti-inflammatory factors (e.g. IL-10, transforming growth factor β), may account for the severity of gastritis in C57BL/6 mice [14, 44, 45]. Consistent with this suggestion, *Ifng* ^−/−^ mice on a C57BL/6 background did not develop gastritis to *H. pylori* infection, while the splenocytes from these animals produced significantly more IL-4 in response to stimulation than those from wild type animals [9]. In the current study, we observed that *Ifng* ^−/−^ BALB/c mice exhibited enhanced Th2 responses to *H. felis* infection, as reflected by increased splenocyte IL-4 production (Fig. 4). Conversely, Th2-type humoral responses (Figs. 2, 3) were unchanged in the *Ifng* ^−/−^ BALB/c mice. Although the reason for this is unclear, a similar lack of concordance between humoral and cytokine responses was reported previously in human subjects given a measles vaccine [46].

T cell responses have been associated with the growth and development of lymphoid follicles which arise at various tissue sites, including the gastric mucosa [6, 40, 47, 48]. These follicles predominantly consist of B cell clones which form premalignant lesions, leading to gastric MALT lymphoma [48–50]. Studies using C57BL/6 mice identified IFN-γ to be an important mediator in the B and T cell interactions required for gastric MALT lymphoma formation [37, 40, 51]. Furthermore, IFN-γ was shown to be highly expressed in the gastric mucosa and to be crucial for B cell clonal expansion and lymphoid follicle formation in C57BL/6 mice infected with *Helicobacter suis* [37]. Interestingly, IL-4 was dispensable in this infection model [37]. The authors also showed that B lymphocytes, and not CD4^+^ T cells, were the main source of gastric IFN-γ and that this cytokine was secreted independently of T cell help [37]. Our study similarly demonstrates a role for IFN-γ in lymphoid aggregate formation in the gastric mucosa of *H. felis*-infected BALB/c mice, which are prone to develop gastric MALT lymphoma [16, 50, 52]. Asides from the potential direct actions of IFN-γ on the immune system, it is possible that its absence in *Ifng* ^−/−^ BALB/c mice resulted in an increased abundance of IL-4-producing Th2 cells, thereby priming naïve B cells and promoting the differentiation of naïve T cells into Th2 cells [35, 52]. Consistent with this suggestion, studies in humans and mice found that B cell proliferation in gastric MALT lymphoma was driven by Th2-polarised T cell responses [52–54]. More work is required to fully elucidate the respective roles of different Th responses in *Helicobacter*-induced gastric MALT lymphoma.

## Conclusions

The fact that IFN-γ was not required for gastritis and bacterial control in BALB/c mice raises an interesting question regarding the role of this cytokine in *Helicobacter* infection. Consistent with previous studies, BALB/c mice appeared to develop less severe inflammation than that typically observed in C57BL/6 animals [18, 24]. Although the differences in pathology observed in BALB/c and C57BL/6 mice may be attributed to their respective Th profiles, it is likely that differences in other immunological and genetic factors between the two murine backgrounds may also be important [3, 20, 23, 30, 35, 43, 55]. This work highlights the importance of considering the genetic background of the host when performing immunological or vaccine studies in *Helicobacter* infection models. Furthermore, the findings reinforce the importance of host genetics on disease outcome, as has been observed in humans with *H. pylori* infection.
